# Predictors of specific anti-*Brucella* antibodies among humans in agro-pastoral communities in Sengerema district, Mwanza, Tanzania: the need for public awareness

**DOI:** 10.1186/s41182-016-0034-5

**Published:** 2016-10-18

**Authors:** Elifuraha B. Mngumi, Mariam M. Mirambo, Sospeter Wilson, Stephen E. Mshana

**Affiliations:** 1 Department of Veterinary Pathology, Sokoine University of Agriculture, P.O. Box 3018, Morogoro, Tanzania; 2 Department of Microbiology and Immunology, Weill Bugando School of Medicine, Catholic University of Health and Allied Sciences, P.O. Box 1464, Mwanza, Tanzania

**Keywords:** *B. abortus*, *B. melitensis*, Brucellosis, Anti-*Brucella* antibodies, Sengerema

## Abstract

**Background:**

Human brucellosis remains to be a neglected zoonotic disease among agro-pastoral communities where livestock rearing is one of the main economic activities. This study was conducted in different agro-pastoral communities in Sengerema district, Mwanza, Tanzania, to determine seroprevalence and predictors of anti-*Brucella* antibodies, information that may influence public awareness on the risk factors and strategies to improve the diagnosis of brucellosis in developing countries.

**Methods:**

A cross-sectional community-based study was conducted between July and September 2008 in ten villages of Sengerema district. Sociodemographic and other related information were collected using a standardized data collection tool. Detection of *Brucella abortus* and *Brucella melitensis* antibodies were done using rapid *Brucella* serum agglutination test. Data were analysed by using STATA version 11.0. Adjusted odds ratios (AOR) were calculated using multivariate logistic regression analysis.

**Results:**

A total of 382 adults were enrolled with the median age of 30 (interquartile range 15–40) years. Males formed the majority of the participants 234 (61.5 %). Overall, seroprevalence of anti-*Brucella* antibodies was found to be 14.1 % (54/382, 95 % CI 10.6–17.5). Seroprevalence of *B. melitensis* was 11 % (42/382) while that of *B. abortus* was found to be 7 % (26/282), *P* = 0.0267. Co-infection of *B. melitensis* and *B. abortus* was observed in 3.6 % (14/382, 95 % CI 1.7–5.4) of participants. On a multivariate logistic regression analysis, male sex (AOR 3.2, 95 % CI 1.3–7.5, *P* = 0.007), touching goat placenta (AOR 2.54, 95 % CI 1.05–6.14, *P* = 0.012) and agro-pastoralist occupation (AOR 2.07, 95 % CI 1.01–4.24, *P* = 0.04) were found to predict *B. melitensis* infection. Males (AOR 3.07, 95 % CI 1.45–6.51, *P* = 0.003) and agro-pastoralists (AOR 2.98, 95 % CI 1.38–6.43, *P* = 0.005) were found to be predictors for specific anti-*Brucella* antibodies.

**Conclusions:**

A significant proportion of the agro-pastoralist male population in agro-pastoral communities in Sengerema district is positive for anti-*Brucella* antibodies. With the decrease incidence of malaria fever, other causes of fever such as *Brucella* spp. should be considered of public health concern in Tanzania especially in agro-pastoral communities.

## Background

Human brucellosis is a global public health concern due to its potential in causing morbidity among human population as well as livestock leading to economic loses. Worldwide, over 500,000 human cases of brucellosis are reported annually [1]. Seroprevalence has been found to vary in different countries across Sub-Saharan Africa [1, 2]. In the tropical countries like Tanzania, *Brucella* infection is endemic especially in agro-pastoral communities [2]. Brucellosis is an occupational disease to slaughterhouse workers, agro-pastoralists, laboratory personnel and veterinarians [3]. Humans can get infection through direct contact with infected farm animals or ingestion of contaminated animal products [4, 5].

*Brucella* infection is characterized by non-specific symptoms including general body malaise, anorexia, fever, back pain, headache, lethargy and many other clinical presentations that often mimic other diseases causing pyrexia such as malaria and typhoid fever [2, 6–8]. Its ability to survive and multiply within immune cells such as macrophages results into chronic debilitating disease with poor prognosis in most of the cases [9]. In addition, the treatment requires multiple antibiotics for prolonged duration. Despite being important, zoonotic disease data regarding the epidemiology of *Brucella* infection among high-risk groups are scarce in developing countries including Tanzania.

Tanzania is a Third World country which has about 51 million people. About 68 % of the Tanzania population is below the poverty line of $1.25 per day [10] and is involved in small-scale agricultural activities. Sengerema district has a total population of 663,034 with the majority of them engaged in agro-pastoralist activities. The poverty and agro-pastoralist activities are risk factors for neglected diseases include brucellosis [11].

This study was conducted to determine seroprevalence and predictors of *Brucella* infection among agro-pastoral communities in Sengerema district. This information may be useful in influencing public awareness on the possible risk factors for infection as well as considering it in diagnosis of febrile illnesses especially in these communities.

## Methods

### Data collection and laboratory procedures

A cross-sectional community-based study was carried out between July and September 2008 in ten agro-pastoral villages in Sengerema district namely Kasungamile, Kabusuli, Lubungo, Magutu, Mami, Ngoma A, Ngoma B, Nyalwambu, Sota and Sota Kaningu. Sociodemographic data and other information related to brucellosis (age, sex, keeping cattle, contact with blood, touching animal placenta, consuming raw milk etc.) were collected using a standardized data collection tool. After obtaining written informed consent, about 4 ml of blood samples were collected using plain Vacutainer tubes (Becton, Dickinson and Company, Nairobi, Kenya) and transported to the Bugando multipurpose laboratory whereby sera were separated and stored in cryovials at −80 °C until processing. Sera were tested for the presence of specific *Brucella melitensis* and *Brucella abortus* antibodies using commercial rapid agglutination test according to the manufacturer’s instructions (Eurocell A/M® Euromedi equip LTD.UK). The Eurocell A® is specific for *B. abortus* and Eurocell M® for *B. melitensis*. The agglutination test has been found to have 95 % sensitivity with specificity of 100 % [12].

### Data management and analysis

Data were entered into a computer using Microsoft Office Excel 2007 and analysed using the STATA version 11 (College Station, Texas, USA). Categorical variables were presented as proportions while continuous variables (age) were summarized as median with interquartile ranges. Stepwise regression model was used to determine factors associated with anti-*Brucella* antibodies. Univariate analysis was done, and factors with *P* value <0.2 were fitted on multivariate logistic regression analysis. Unadjusted odds ratio (UAOR), adjusted odds ratio (AOR) and 95 % confidence interval (CI) were noted. *P* value of <0.05 was considered statistically significant.

## Results

### Baseline characteristics

A total of 382 participants from agro-pastoral communities in different villages of Sengerema district were recruited with the median age of 30 (interquartile range (IQR) 15–40) years. Males 234 (61.5 %) formed the majority of the study population. Out of 382 participants, 117 (30.6 %) and 245 (64.1 %) were students and agro-pastoralists, respectively. A total of 294 (77 %) participants were found to keep cattle (Table 1).

**Table 1:** Baseline characteristics of 382 adult participants from Sengerema district

Characteristics	Frequency/median	Percent
Age	30 (IQR 15–40)	
Sex		
Female	148	38.5
Male	234	61.5
Occupation		
Students	117	30.6
Agro-pastoralists	245	64.1
Others^a^	20	5.2
Keep cattle		
No	88	23.0
Yes	294	77.0

^a^Businessmen, teachers and fishermen

### Prevalence of specific anti-*Brucella* antibodies

Overall, seroprevalence of brucellosis was found to be 14.1 % (54/382, 95 % CI 10.6–17.5). Seroprevalence of *B. melitensis* antibodies was found to be 11 % (42/382, 95 % CI 7.8–14.1) while for *B. abortus* was 7 % (26/282, 95 % CI 4.4–9.5), *P* = 0.026. Seroprevalence of anti-*Brucella* antibodies indicative of co-infection with both *B. melitensis* and *B. abortus* was found to be 3.6 % (14/382, 95 % CI 1.7–5.4).

### Factors associated with the presence of specific anti-*Brucella* antibodies

The median age of participants who tested positive for *B. melitensis* antibodies was 30 (IQR 19–40) years compared to 30 (IQR 15–40) years for those tested negative (*P* = 0.489). Males had significantly higher *B. melitensis* antibodies than females (14.5 vs. 5.4 %, *P* = 0.008). On multivariate logistic regression analysis, male sex (AOR 3.2, 95 % CI 1.3–7.5, *P* = 0.007), touching goat placenta (AOR 2.54, 95 % CI 1.05–6.14, *P* = 0.012) and agro-pastoralist occupation (AOR 2.07, 95 % CI 1.01–4.24, *P* = 0.04) were found to predict *B. melitensis* infection (Table 1).

Regarding *B. abortus*, there was no significant difference between male and female sex (8.5 vs. 4.1 %, *P* = 0.097). On univariate logistic regression analysis, agro-pastoralists were more likely to contract *B. abortus* infection than students (OR 7.3, 95 % CI 1.71–31.51, *P* = 0.007). Only occupation (OR 7.44, 95 % CI 1.42–38.9, *P* = 0.02) remained significant predicting *B. abortus* infection when adjusted for age, touching placenta, touching goat placenta and blood splash (Table 2).

**Table 2:** Factors associated with *B. melitensis* seropositivity among 382 adults from agro-pastoral communities in Sengerema district

Characteristics	*B. melitensis* seropositivity	Unadjusted OR (95 % CI)	*P* value	Adjusted OR (95 % CI)	*P* value
Age (years)*	42 (IQR 19–40)	1.0 (0.98–1.02)	0.628		
Sex					
Female (148)	8 (5.4 %)	1			
Male (234)	34 (14.5 %)	2.9 (1.33–6.62)	0.008	3.2 (1.3–7.5)	0.007
Occupation					
Students (117)	10 (8.5 %)	1			
Agro-pastoralists (245)	32 (13.1 %)	1.9 (0.90–4.01)	0.088	2.07 (1.01–4.24)	0.04
Keep cattle					
No (88)	9 (10.2 %)	1			
Yes (294)	33 (11.2 %)	1.1 (0.5–2.41)	0.793		
Touch placenta					
No (289)	28 (9.7 %)	1			
Yes (93)	14 (15.1 %)	1.65 (0.82–3.29)	0.153	0.75 (0.34–1.67)	0.493
Touch goat placenta					
No (341)	32 (9.4 %)	1			
Yes (41)	10 (24.4 %)	3.11 (1.4–6.9)	0.005	2.59 (1.25–6.38)	0.012
Blood splash					
No (360)	37 (10.3 %)	1			
Yes (22)	5 (22.7 %)	2.5 (0.89–7.4)	0.079	1.5 (0.47–4.98)	0.47

*Median

Overall, males (18.4 vs. 7.4 %, *P* = 0.004) and agro-pastoralists (18.0 vs. 8.5 %, *P* = 0.005) had significantly higher rates of *Brucella*-specific antibodies than females and students, respectively (Table 3). These factors were independently found to be associated with *Brucella* infection with either of the two species (Table 4). On multivariate logistic regression analysis, male sex (OR 3.07, 95 % CI 1.45–6.51, *P* = 0.003) and being agro-pastoralist (OR 2.98, 95 % CI 1.38–6.43, *P* = 0.005) were found to predict the presence of *Brucella*-specific antibodies when adjusted to touching placenta and touching goat placenta.

**Table 3:** Factors associated with *B. abortus* seropositivity among 382 adults from agro-pastoral communities in Sengerema district

Characteristics	*B. abortus* seropositivity	Unadjusted OR (95 % CI)	*P* value	Adjusted OR (95 % CI)	*P* value
Age (years)*	30 (IQR 23–47)	1.02 (0.99–1.04)	0.069	0.98 (0.96–1.01)	0.489
Sex					
Female (148)	6 (4.05 %)	1			
Male (234)	20 (8.5 %)	2.2 (0.87–5.64)	0.097	2.1 (0.753–6.14)	0.152
Occupation					
Students (117)	2 (1.7 %)	1			
Agro-pastoralists (245)	24 (9.8 %)	7.3 (1.71–31.51)	0.007	7.44 (1.42–38.9)	0.017
Keep cattle					
No (88)	4 (4.55 %)	1			
Yes (294)	22 (7.5 %)	1.69 (0.57–5.07)	0.342		
Touch placenta					
No (289)	14 (4.84 %)	1			
Yes (93)	12 (12.9 %)	2.9 (1.29–6.5)	0.010	1.35 (0.52–3.4)	0.529
Touch goat placenta					
No (341)	20 (5.87 %)	1			
Yes (41)	6 (14.63 %)	2.7 (1.04–7.3)	0.042	1.76 (0.59–5.27)	0.307
Blood splash					
No (360)	22 (6.11 %)	1			
Yes (22)	4 (18.2 %)	3.4 (1.06–10.9)	0.034	1.89 (0.52–6.88)	0.329

*Median

**Table 4:** Factors associated with *Brucellosis* among 382 adults from agro-pastoral communities in Sengerema district

Characteristics	*Brucellosis*	Unadjusted OR (95 % CI)	*P* value	Adjusted OR (95 % CI)	*P* value
Age (years)*	30 (IQR 20–44)	1.01 (0.99–1.02)	0.227		
Sex					
Female (148)	11 (7.43 %)				
Male (234)	43 (18.38 %)	2.80 (1.39–5.63)	0.004	3.07 (1.45–6.51)	0.003
Occupation					
Students (117)	10 (8.5 %)	1			
Agro-pastoralists (245)	44 (18.0 %)	2.7 (1.35–5.72)	0.005	2.98 (1.38–6.43)	0.005
Keep cattle					
No (88)	11 (12.5 %)				
Yes (294)	43 (14.6 %)	1.20 (0.59–2.43)	0.616		
Touch placenta					
No (289)	35 (12.1 %)				
Yes (93)	19 (20.4 %)	1.86 (1.01–3.45)	0.048	0.86 (0.42–1.75)	0.687
Touch goat placenta					
No (341)	43 (12.6 %)				
Yes (41)	11 (26.8 %)	2.5 (1.18–5.44)	0.016	2.10 (0.94–4.71)	0.069
Blood splash					
No (360)	49 (13.6 %)				
Yes (22)	5 (22.7 %)	1.87 (0.65–5.30)	0.240		

*Median

## Discussion

Brucellosis is one of the public health concerns due its potential in causing human infection and economic loses among agro-pastoralists [13]. Despite having impact on livelihoods, it is one of the neglected tropical diseases in most of the developing countries. Despite this study being conducted 8 years ago, the situation now is comparable to the time the study was conducted. In the present study, a significant proportion of agro-pastoralists was infected with *B. melitensis* which is comparable to previous studies [14–19]. On the contrary, the prevalence observed in this study is lower as compared to what has been reported earlier in Nigeria, Libya and Kenya [13, 20, 21]. The difference could be attributed by the fact that these previous studies were done among febrile patients and butcher workers which are among high-risk groups for brucellosis. In addition, the seroprevalence of *B. abortus* in this study was found to be significantly lower than that of *B. melitensis* which is in agreement with previous study [22].

Among the risk factors assessed, male sex, agro-pastoral occupation and touching goat placenta were found to be associated with *B. melitensis* infection among agro-pastoral communities which is consistent to previous reports [14, 20, 23–27]. Meanwhile, agro-pastoralism was the only factor found to predict *B. abortus* infection while male sex and agro-pastoralism were found to predict the presence of *Brucella* antibodies. The finding of touching goat placenta predicting *B. melitensis* infection confirms its presence in goat and sheep as the main host [28]. On the other side, male sex and agro-pastoralism were found to predict brucellosis as previously observed [29, 30]. Predominance of male sex and agro-pastoralism could be explained by the traditional roles of males in these communities whereby they are much more involved in livestock care as compared to female counterparts. Other studies have documented female sex to be risk factors for brucellosis, and this could be explained by female involvement in agro-pastoral activities, signifying the importance of occupation as the major risk factor for contracting *Brucella* infection [31].

One of the major limitations of the study is the recall bias; majority of the study participants might have forgotten the previous risk behaviour regarding brucellosis. The other limitation is inability to distinguish past and present infections. Despite these limitations, the information the data obtained will help in improving the diagnosis of other causes of fever in developing countries.

## Conclusions

There is high seroprevalence of anti-*Brucella* antibodies among agro-pastoralists in Tanzania. With a decreased trend in malaria infections, diagnosis of other causes of febrile illnesses should be considered in these agro-pastoral communities.

## Abbreviations

AOR: Adjusted odds ratio
BMC: Bugando Medical Centre
CI: Confidence interval
CUHAS: Catholic University of Health and Allied Sciences
IQR: Interquartile range
UAOR: Unadjusted odds ratio
VIC: Veterinary Investigation Centre
